# Rays Sting: The Acute Cellular Effects of Ionizing Radiation Exposure

**Published:** 2016-05-16

**Authors:** A Franco, M Ciccarelli, D Sorriento, L Napolitano, A Fiordelisi, B Trimarco, M Durante, G Iaccarino

**Affiliations:** 1 Department of Advanced Biomedical Sciences, “Federico II” University of Naples, Italy; 2 Department of Medicine and Surgery, University of Salerno, Italy; 3 IBB-CNR, Naples, Italy; 4 Trento Institute for Fundamental Physics and Applications, Trento, Italy

**Keywords:** Reactive Oxygen species, signal transduction, ionizing radiations, Mitochondria

## Abstract

High-precision radiation therapy is a clinical approach that uses the targeted delivery of ionizing radiation, and the subsequent formation of reactive oxygen species (ROS) in high proliferative, radiation sensitive cancers. In particular, in thoracic cancer ratdiation treatments, can not avoid a certain amount of cardiac toxicity. Given the low proliferative rate of cardiac myocytes, research has looked at the effect of radiation on endothelial cells and consequent coronary heart disease as the mechanism of ratdiation induced cardiotoxicity. In fact, little is known concerning the direct effect of radiation on mitochondria dynamis in cardiomyocyte. The main effect of ionizing radiation is the production of ROS and recent works have uncovered that they directly participates to pivotal cell function like mitochondrial quality control. In particular ROS seems to act as check point within the cell to promote either mitochondrial biogenesis and survival or mitochondrial damage and apoptosis. Thus, it appears evident that the functional state of the cell, as well as the expression patterns of molecules involved in mitochondrial metabolism may differently modulate mitochondrial fate in response to radiation induced ROS responses. Different molecules have been described to localize to mitochondria and regulate ROS production in response to stress, in particular GRK2. In this review we will discuss the evidences on the cardiac toxicity induced by X ray radiation on cardiomyocytes with emphasis on the role played by mitochondria dynamism.

## I. INTRODUCTION

Ionizing irradiation is defined as the transport of energy through the space. In biomedicine the effects of irradiation are studied to evaluate the modifications on cells and tissues. Ionizing irradiation for example is a cause of cancer by inducing modifications of the genetic information in individual cell^1^, ^2^. At the same time, radiation is also applied for treatment of cancer with purpose of killing cancer cells^3^. About 60% of all cancer diseases are cured by radiotherapy alone or in combination with surgery^4^. The use of radiation in tumor therapy represents a compromise between maximal damage of tumor cells and the minimal deleterious effects for healthy tissues. For this purpose, high-precision radiation therapy has been developed to minimize damage of the surrounding normal tissues^5^, ^6^. This approach uses the delivery of ionizing radiation with selective formation of reactive oxygen species (ROS) in the targeted tissue^7^, a biological effect that can be relived starting within milliseconds after exposure. The specific subcellular alterations induced by radiation involve mainly plasma and mitochondria membrane with following increased production of ROS and possibly alteration of the mitochondrial function^8^, ^9^. However, even though this intensity-modulated radiotherapy can reduce the exposure to the normal tissues, a certain amount of radiation is still delivered in the area surrounding the neoplasia. The consequence of this damage can be divided in early and late reactions, based on their occurrence within hours (acute exposition)^10^ or days/months/years after therapy (chronic exposition)^11^. The effects of ionizing radiation used in radiotherapy on different tissues are of a particular interest for the clinical consequences at the cardiovascular system. Thoracic radiotherapy is among the most frequent applications used for treatment of mediastina neoplasia, such as breast cancer or Hodgkin Lymphoma, and it is frequently associated to a clinically relevant cardiac toxicity, occurring as late reactions^12^.

Several studies have pointed out the effects of radiation on vascular endothelial cells^13^–^15^ but recently it has been observed that radiation can also directly affects the cardiomyocytes^16^ and other cardiac structures leading to cardiomyopathy^17^, valves heart disease and conduction abnormalities^18^. However, the knowledge about the direct effects of radiation on the myocardium is still poor, as related to the effects on the single cardiomyocyte and the specific molecular alteration produced^19^. Mitochondria are considered the cardiomyocytes powerhouse and are at the same time the major source of ROS^20^. Considering the relevance of mitochondria for cardiac functions, it is possible to speculate that the deleterious effects of a chronic irradiation could relate to the dysfunction of this organelle^21^. In this review we will discuss the latest evidences on the cardiac toxicity induced by ionizing radiation (X-ray) on cardiomyocytes with emphasis on the role played by mitochondria.

## II. PHYSICAL AND BIOLOGICAL EFFECTS OF X-RAY

### A. Physical Properties Of X-Rays

A radiation is defined as the transport of energy in space, which is then transferred to the matter. The radiation is quantified and measured in elettronVolt (eV). According to the Electromagnetic Spectrum a radiation can be divided in Non-Ionizing Radiation (< 10 eV) or Ionizing Radiation (> 10 eV). When the radiation reaches the body, it excites the atoms of the molecules of biological tissues. Related to the absorbed dose, the biological consequences caused by ionizing radiation can change depending on the nature of radiation involved^22^: α particle, β particle and X- Y Ray, where α and β are constituent of corpuscular radiation^23^, while X and Y are electromagnetic radiation. Specifically, X-rays are classified as an electromagnetic, indirect ionizing radiation because it produces secondary electrons with high kinetic energies. These electrons in turn can cause damage in the absorbing matter. The electron vacancy in the atomic shell, caused by an ejection, is filled with an electron from an outer shell subsequently leading to the emission of a photon. A typical interaction between an X-ray photon and a water molecule is^24^:

$${\text{H}}_{2}0\to {\text{H}}_{2}0^{+}+{\text{e}}^{-}$$

Where H_2_0^+^ is a highly reactive ion radical. The reaction between H_2_0^+^ and water molecule produces the hydroxyl radical H0^−^

$${\text{H}}_{2}0^{+}+{\text{H}}_{2}0\to {\text{H}}_{3}0^{+}+\text{H}0^{-}$$

Which is a highly reactive oxygen species and it is responsible for the biological effects of X-ray. ROS accumulation leads to apoptotic cell death^25^ and is associated with the accumulation of damage that cannot be recovered in mitochondria and Nucleus. For example, typical features induced by radiation involve the Nucleus with induction of point mutations^26^. However, when the apoptotic process does not eliminate a transformed cell, cytogenetic damages such as translocations pass to daughter cells^27^. Thus, referring to the entire organism, radiation-induces DNA damages may lead to cancer or to hereditary diseases according to the specific cell damaged.

In general, X-ray biological effects are typically divided into two categories. The first category consists of exposure to high doses of radiation over short periods of time producing acute or short-term effects^28^. The second category represents exposure to low doses of radiation over an extended period of time producing chronic or long-term effects. High doses tend to kill cells, while low doses tend to damage or change the functions of the several substructures. High doses can kill so many cells that tissues and organs are damaged or even destroyed^29^. This in turn causes a rapid whole body response called the Acute Radiation Syndrome (ARS).

Low doses spread out over long periods of time and do not cause an immediate and clinical evident problem. The effects of low doses of radiation occur primarily at cellular level, and the effects may not be observed for many years. Here we will describe in details the biological effects of low and chronic exposure to X-ray.

### B. Nuclear Effects Of X-Ray

There are three general categories of effects resulting from exposure to low doses of radiation. These are: 1) Genetic, when the effect is suffered by the offspring of the individual exposed. 2) Somatic^30^, which primarily involves the individual exposed. Since cancer is the primary result, it is also called the Carcinogenic Effect. 3) In-Utero^31^,^32^, mistakenly considered as a genetic consequence of radiation exposure, because the effect, suffered by a developing embryo/fetus, is seen after birth.

In this contest DNA plays a key role in the response to radiation. Since the information on both strands of the DNA molecule is complementary, all injuries affecting only one side of the DNA double strand can be easily repaired by using the information on the intact strand as a template^33^. Therefore, double strand breaks (DSB) are generally considered as the critical event for the induction of lethal lesions^34^, ^35^. Mammalian cells are in general able to recognize and repair damage to DNA for a certain extent^36^. The efficiency of these repair processes depends on the complexity of the damage induced^37^. For example, single strand breaks can be easily repaired during the replication cycle, when the double strand has to be opened on one strand to allow the access of replication and reparative proteins to the DNA. But with increasing complexity, damage becomes more difficult to repair, and this might enhance the probability that this process cannot be accomplished correctly, leaving a partially repaired or modified DNA molecule. DNA damage can be induced by radiation in two different ways. On the one hand, radiation leads to ionizing events in the DNA molecule itself, subsequently leading to breakage of molecular bonds and disruption of one or both strands of the DNA. These events are termed ‘direct effect’. On the other hand, radiation leads to the production of, highly reactive OH-radicals by radiolysis of the water molecules surrounding the DNA molecule. These radicals are able to migrate over distances of a few nanometers during their lifetime and are thus capable of damaging the DNA molecule of distant cells. This action is termed “indirect effect”. However, these types of lesions do not necessarily occur separately, but instead, depending on the dose level, combinations of both effects can lead to DNA damage.

As mentioned above, X-ray are mainly indirectly ionizing because they do not directly induce chemical damage but produce secondary electrons with high kinetic energies such as hydrogen (H) and hydroxyls (OH). These fragments may recombine or may interact with other fragments or ions to form compounds, such as water, which would not harm the cell. However, they also combine to form toxic substances, such as hydrogen peroxide (H2O2), which can contribute to the destruction of the cellular structure, indicating that part of the toxic effects of X-ray are mediated by ROS.

#### C. Ros In The Intracellular Signaling: Only A Mediator Of X-Ray Induced Damage?

The perceived roleof ROS in regulation of the cellular physiology has changed in the recent years. Indeed, if on a side they are considerate as detrimental for cell survival, they also showimportant physiological roles and act as part of the intracellular signaling, promoting beneficial cellular process as mitohermesis, defined as replacement and organization of the mitochondrial network. The list of intracellular targets of ROS is growing rapidly and several targets have been identified: 1) The mitogen-activated protein kinase (MAPK) family, also known as extracellular signal– regulated kinases (ERKs), is activated by exogenous and endogenous H_2_O_2_ in cells stimulated with growth factors^38^. ERKs are important mediators of proliferation and their activation is implicated in vascular endothelial growth factor (VEGF)–mediated Endothelial Cell survival^39^. 2) Akt/PKB kinase, is known to be involved in anti-apoptotic signaling and it is also regulated by ROS^40^. Specifically, Akt is activated in the vascular endothelium through increased level of H2O2 by the membrane NAD(P)H oxidase which in turn is activated by shear stress. 3) Activation of the transcription factor NF-κB, which is associated with Endothelial Cell dysfunction and vascular inflammation, is also regulated by ROS. These evidences suggest ROS as a specific cellular messenger able to promote either cellular survival and adaptation or apoptosis, according to the specific characteristics of the stressors. This knowledge may also change the view of ionizing radiation in clinic and therapy. The effects of ROS induced by radiation are different according to the dose and time of exposure^41^ ^42^ and considering that the major ROS production is given by mitochondria^43^, it would not be surprising that the cellular effects induced by X-ray depend on modification of mitochondrial function in a dose and time dependent manner^44^. Indeed, recent works have uncovered that ROS directly participates to pivotal cell function like mitochondrial quality control^45^–^47^.

Mitochondria occupy a substantial portion of the cytoplasmatic volume of eucaryotic cells, in particular cardiomyocytes. Each mitochondria is bounded by two highly specialized membranes, which have very different function: 1) A outermembranes (O.M.) that contains many copies of transport protein called porin (MPTP)^48^ ; 2) Inner membranes (IM) that is usually highly convoluted, forming a series of unfolding, know as cristae, that project into the matrix. Together they create two separate mitochondrial compartments: the internal matrix (IM) and a narrower intermembrane space. In the mitochondria, the metabolism of sugars is completed, and the energy released is harnessed so efficiently that about 30 molecules of ATP are produced for each molecule of glucose oxidized. Oxidative phosphorylation is made possible by the close association of the electron carriers with protein molecules. The electron transport chain (ETC) resides on the inner membrane and it is composed by a series of protein complex that transfer electrons from electron donor to electron acceptors via redox reactions, and couples this electron transfer with the transfer of protons (H+ ions) across the membrane. The proteins guide the electrons along the respiratory chain so that the electrons move sequentially from one enzyme complex to another without short circuits. Most importantly, the transfer of electrons is coupled to oriented H^+^ uptake and release, as well as to allosteric changes in energy-converting protein pumps^49^. The net result is the pumping of H^+^ across the inner membrane from the matrix to the inter-membrane space, driven by the energetically favorable flow of electrons. This movement of H+ has two major consequences:

- Generates a Ph gradients, and
- A voltage gradient, which is defined as the mitochondrial membrane potential.

**Figure 1:**
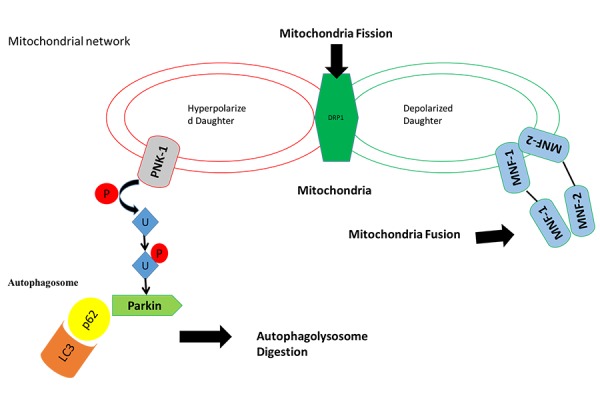
In response to stress a part of mitochondria loss its mitochondrial potential membranes and Drp-1 induces (mitochondrial fission), consequentially PINK-1 is exposes on mitochondria damage and it drives mitochondria degradation trough phosphorilation of ubiquitination and activation of ubiquitine ligase Parkin (autophagosome digestion). The mitochondria has not lost its membrane potential activates the mitochondrial fusion through mitofusin 1, mitofusin 2 and Opa-1 with other part of mitochondria that has preserved its mitochondria potential membrane to form a new functional mitochondria (Mitochondria fusion).

A reduction of mitochondrial potential membrane is indicator of mitochondrial dysfunction and induces the mitochondria degradation and removal^50^. This introduces the concept of “Mitochondrial dynamics”^51^ which refers to organelle fission, fusion, and subcellular translocation and this process is fundamental, in response to stress, to recover the mitochondrial damage and cellular survival^52^. In response to stress, a portion of damaged mitochondria loses its membrane potential and cleaved from DRP-1 (Dynamin Related Protein)^53^ generating two mitochondria daughters that are destined to elimination (mitophagy) or recovering (mitochondrial fusion). In response to the reduction of mitochondrial membranepotential and DRP-1 activation, the portion of injured mitochondria expresses PINK-1, a mitochondrial serine/threonine protein kinase^54^. PINK-1 is intimately involved in “mitochondrial quality control” (QC) allowing targeting mitochondria to elimination^50^. Healthy mitochondria maintain a membrane potential and import PINK1 into the inner membrane where it is cleaved by PARL. Severely damaged mitochondria lack sufficient membrane potential to import PINK1, which then accumulates on the outer membrane. PINK-1 phosporylates the ubiqutin and induces Parkin on Ser65 which is then allowed to move to mitochondria^55^, ^56^. Parkin is component of an E3 ubiquitin ligase complex, that together with other molecules such as P62, LC3-I/II, forms an ubiquitinproteasome system designated to the removal and degradation of entire or part of mitochondria^57^, ^58^. The mitochondria that has not lost its membrane potential activates the mitochondrial fusion^59^, ^60^ through mitofusin 1, mitofusin 2^61^–^63^ and Opa-1^64^ allowing recovering of the two “daughter” mitochondria (Fig. 1).

Together mitochondria fusion and fission regulates the mitochondrial dynamism^20^, ^65^ and promote cellular survival in response to stress^66^. In summary, the mitochondrial dynamism is implicated in structural remodeling of mitochondria network and in homeostatic maintenance of mitochondrial DNA stability and respiratory function preventing or propagating programmed cell death, in particular in response to stress^67^. The combined effects of continuous fusion and fission give rise to mitochondrial networks, to preserve organ functions.

In this context it is interesting to introduce the effect of X-ray exposure on the mitochondrial functions^43^. X-ray exposition can directly damage mitochondrial membrane^68^, ^69^ and/or indirectly through increasing ROS production^70^. In both cases, the consequence is the reduction of mitochondrial potential membranes and thus activation of fission/fusion process^20^. As described above, these mechanisms are fundamental for cell survival and maintenance within physiologic conditions, therefore increased ROS production induced by X-ray can even promote the renewing of the mitochondria^71^.However, when the amount of accumulated ROS, as it happens with chronic exposure to low doses of X-ray, overcomes mechanisms of repair and it activates pro-apoptotic events and programmed cell death^72^.

## II. X-RAY AND CARDIOVASCULAR EFFECTS: ROLE OF MITOCHONDRIA

Cardiovascular disease and cancer are the two leading causes of mortality and morbidity worldwide^73^. These two pathological conditions frequently run together in presence of cancer disease treated with radiotherapy. Radiation therapy (RT) has evolved to be a cornerstone for treatment of various types of cancers (about 50% of patients with cancer are treated with radiotherapy). Chronic mediastina radiation can involve the pericardium, myocardium, valves and coronary vessels with pericardium being most frequently involved^74^, ^75^. Thus, it is not surprising that for specific type of cancer like Hodgkin lymphoma (HL) an important cause of death is represented by cardiovascular complications^76^ ^74^, ^77^. Several studies show that X-ray produce vascular endothelium abnormalities with resulting telangectasia, thrombotic and inflammatory alteration^78^ in large vessels, which can finally result in coronary and carotid artery disease^79^, ^80^. The effects of X-ray are time and dose dependent and include a wide number of effects, starting from endothelial dysfunction, lipid and inflammatory cells infiltration till formation of atherosclerotic plaque^81^, ^82^. In particular, in animal model treated with elevated cholesterol-diet, the radiation stress abridges atherosclerosis, due to an increased macrophages infiltration into the arterial wall^83^, ^84^.

The effects of X-ray on myocardium^85^ is less known but it is emerging that this radiation can interfere with mitochondria functions, with relevant consequences considering the heart as a dynamic organ with an abundant number of mitochondria^86^. Therefore, mitochondria damage can be pivotal in fostering the cardiac alterations produced by chronic irradiation^87^.

**Fig 2:**
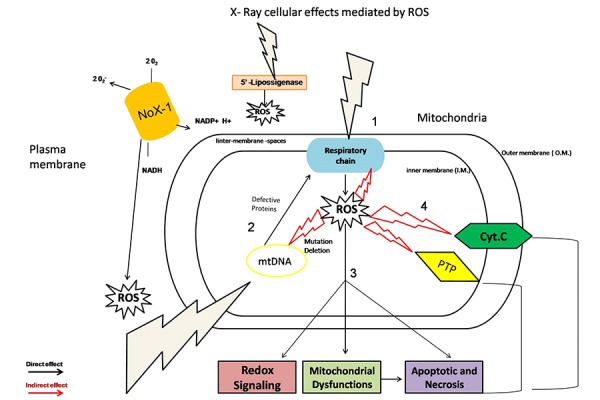
X-Ray cellular effects mediated by ROS. X –rays on plasma membrane induce an increase of ROS through activation of NOX1 and (5’ lipossigenase). X-ray can target to mitochondria bothe directly and indirectly: through increased ROS production: 1) the compounds of respiratory chain. 2) mtDNA generating trough mutation and deletion defective proteins to respiratory chain. 3) Alteration of redox signaling and mitochondrial dysfunctions that induces necrosis and apoptotic events. 4) Release of Cytocrome C outer mitochondria membranes into cytosolic fractions and formation of mPTP channel to induce respectively apoptotic and necrosis events.

Role of Mitochondria in mediating the effects of X-ray on cellular survival have been related in part to the direct effect on the external mitochondrial membrane and to the phenomenon of oxidative stress (Fig 2).

However the specific molecular mechanisms are far to be elucidated. For future investigations we may start from some observations: 1) The heart is an organ with an high energy demand, needed to accomplish its contractile function^88^ 2) A preserved mitochondrial function is indeed fundamental for cardiac function and an extensive literature is now available showing the role of mitochondrial dysfunction in the setting of chronic diseases^89^, like cardiac aging^90^, ^91^, ischemia or heart failure^92^–^96^. 3) X-ray used for treatment of LH can lead to a cardiac eccentric remodeling with systolic dysfunction, which is a phenotype observed in many other cardiac pathological conditions^85^, ^95^, ^97^. Therefore the molecular mechanisms and mitochondrial process involved in myocardial injury induced by X-ray could not be so different to those observed in other cardiac diseases^98^. It is possible speculate that X-ray chronic exposure as observed in patients treated with radiotherapy, the accumulate damage of mitochondria cannot be further recovered, as seen in human and animal model of cardiac aging and heart failure^99^. Mitochondria are dynamic organelles where mechanism of mitogenesis and fission/fusion allows either produce new organelle or recover damaged mitochondria^100^. However, it is also part of a network, with functional connection with endoplasmatic reticulum^101^, nucleus and cytosol and it is exposed to the influence of several intracellular signaling that regulate mitochondria responses to the external stress. For example, the master regulator of mitochondrial biogenesis is PGC-1α, which is not a resident mitochondrial protein^102^. This molecule lacks DNA-binding activity but interacts with and co-activates numerous transcription factors including NRFs on the promoter of mtTFA. PGC-1α is enriched in tissue with high oxidative activity-like heart and brown adipose tissue and it is rapidly induced under conditions of increased energy demand and stress such as cold, exercise, and fasting. PGC-1α can be regulated by different intracellular signaling (Thyroid hormone (TH), nitric oxide synthase (NOS/cGMP), p38 mitogen-activated protein kinase (p38MAPK), sirtuines (SIRTs), calcineurin, calcium-calmodulin-activated kinases (CaMKs), adenosine-monophosphate-activated kinase (AMPK), cyclin-dependent kinases (CDKs), and β-adrenergic stimulation (β/cAMP) through post-translational modifications, rendering it as the integrative step for the different conditions^103^, ^104^.

Mechanisms of fission/fusion are also governed by non-mitochondrial molecules and signalling. These pathways are activated in stress conditions, such as H2O2, UV and ischemia, with following modifications of the mitochondrial morphology and dynamic^105^, ^106^. We observed similar mitochondrial modificationsin cells exposed to a single dose of X-ray irradiation, where the initial mitochondrial damage and disarrangements is there after recovered in a relative short period of time (about 8 hour), suggesting involvement of the mitochondrial quality control mechanism^107^. Specific cytosol molecules could be triggered: for example Mitogen-activated protein (MAP) kinase cascade member extracellular-signal-regulated kinase (ERK) has been shown to phosphorylate the pro-fusion protein mitofusin (MFN) 1, modulating its participation in apoptosis and mitochondrial fusion^107^. Moreover, ERK signalling can also indirectly modulates other proteins such as HSP90 and GRK2^108^, which have been also demonstrated to localize at mitochondria^109^, ^110^. GRK2 function in mitochondria is still debated and controversial, since it appears to act both as pro-death kinase and as protective in terms of improved biogenesis after ischemi are perfusion injury^111^. Our recent data, however, have shown that X-ray irradiation promotes GRK2sub-cellular localization into mitochondria in a time dependent manner and GRK2 knockdown affects the mechanisms of mitochondrial recovering, thus suggesting a key role in the mechanism of quality control.

## III. SUMMARY AND CONCLUSIONS

Hearts exposed to radiotherapy can accumulate mitochondrial damage that cannot be further recovered through the mechanisms of mitogenesis and/or quality control. The molecular mechanisms are probably not so distant from what observed in other conditions, where the reduced tolerance to stress is associated with impairment of pathways participating to the mitochondria-ER-nucleus network, fundamental to promote cellular adaptation to the stress. Models of heart failure post myocardial infarction, aging or diabetes^112^ have shown the central role of mitochondrial dysfunction in the progression of the disease and the related discoveries are leading to specific approaches aimed to recover mitochondrial dynamic and functions^107^. Therefore, a new challenge for the years to come will be the identification of the specific role played by this organelle in cardiac alterations induced by X-ray stress and so the development of new approaches to enhance and/or preserve mitochondrial functions and network during chronic radiation stress.

## References

[1] Maity A, McKenna WG, Muschel RJ (1994). The molecular basis for cell cycle delays following ionizing radiation: A review. Radiotherapy and oncology : journal of the European Society for Therapeutic. Radiology and Oncology.

[2] Hall EJ, Freyer GA (1991). The molecular biology of radiation carcinogenesis. Basic life sciences.

[3] Delaney G, Jacob S, Featherstone C, Barton M (2005). The role of radiotherapy in cancer treatment: Estimating optimal utilization from a review of evidence-based clinical guidelines. Cancer.

[4] Tennvall J, Lundell G, Wahlberg P, Bergenfelz A, Grimelius L, Akerman M, Hjelm Skog AL, Wallin G (2002). Anaplastic thyroid carcinoma: Three protocols combining doxorubicin, hyper fractionated radiotherapy and surgery. British journal of cancer.

[5] Griffin TW, Wambersie A, Laramore G, Castro J (1988). International clinical trials in radiation oncology. High let: Heavy particle trials. International journal of radiation oncology, biology, physics.

[6] McMahon SJ, McGarry CK, Butterworth KT, Jain S, O’Sullivan JM, Hounsell AR, Prise KM (2015). Cellular signalling effects in high precision radiotherapy. Physics in medicine and biology.

[7] Yamaguchi M, Kashiwakura I (2013). Role of reactive oxygen species in the radiation response of human hematopoietic stem/progenitor cells. PloS one.

[8] Sag CM, Wolff HA, Neumann K, Opiela MK, Zhang J, Steuer F, Sowa T, Gupta S, Schirmer M, Hunlich M, Rave-Frank M, Hess CF, Anderson ME, Shah AM, Christiansen H, Maier LS (2013). Ionizing radiation regulates cardiac ca handling via increased ros and activated camkii. Basic research in cardiology.

[9] Zhou L, Aon MA, Liu T, O’Rourke B (2011). Dynamic modulation of ca2+ sparks by mitochondrial oscillations in isolated guinea pig cardiomyocytes under oxidative stress. Journal of molecular and cellular cardiology.

[10] Anno GH, Baum SJ, Withers HR, Young RW (1989). Symptomatology of acute radiation effects in humans after exposure to doses of 0.5–30 gy. Health physics.

[11] Chinnaiyan AM, Prasad U, Shankar S, Hamstra DA, Shanaiah M, Chenevert TL, Ross BD, Rehemtulla A (2000). Combined effect of tumor necrosis factor-related apoptosis-inducing ligand and ionizing radiation in breast cancer therapy. Proceedings of the National Academy of Sciences of the United States of America.

[12] McReynolds RA, Gold GL, Roberts WC (1976). Coronary heart disease after mediastinal irradiation for hodgkin’s disease. The American journal of medicine.

[13] Azimzadeh O, Sievert W, Sarioglu H, Merl-Pham J, Yentrapalli R, Bakshi MV, Janik D, Ueffing M, Atkinson MJ, Multhoff G, Tapio S (2015). Integrative proteomics and targeted transcriptomics analyses in cardiac endothelial cells unravel mechanisms of long-term radiation-induced vascular dysfunction. Journal of proteome research.

[14] Wang J, Boerma M, Fu Q, Hauer-Jensen M (2007). Significance of endothelial dysfunction in the pathogenesis of early and delayed radiation enteropathy. World journal of gastroenterology : WJG.

[15] Hopewell JW, Calvo W, Jaenke R, Reinhold HS, Robbins ME, Whitehouse EM (1993). Microvasculature and radiation damage. Recent results in cancer research. Fortschritte der Krebsforschung. Progres dans les recherches sur le cancer.

[16] Friess JL, Heselich A, Ritter S, Haber A, Kaiser N, Layer PG, Thielemann C (2015). Electrophysiologic and cellular characteristics of cardiomyocytes after x-ray irradiation. Mutation research.

[17] Kursova LV, Konoplyannikov AG, Kal’sina S, Baboyan SB (2014). Allogenic cardiomyoblasts raised from human mesenchymal stem cells in the therapy of radiation cardiomyopathy and pericarditis: Case report. Bulletin of experimental biology and medicine.

[18] Ong DS, Aertker RA, Clark AN, Kiefer T, Hughes GC, Harrison JK, Bashore TM (2013). Radiation-associated valvular heart disease. The Journal of heart valve disease.

[19] Wieshammer S, Dreyhaupt J, Muller D, Momm F, Jakob A, Freund U (2013). Cardiotoxicity and cancer therapy: Treatment-related cardiac morbidity in patients presenting with symptoms suggestive of heart or lung disease. Oncology.

[20] Dorn GW (2015). Mitochondrial dynamism and heart disease: Changing shape and shaping change. EMBO molecular medicine.

[21] Wu L, Tan JL, Wang ZH, Chen YX, Gao L, Liu JL, Shi YH, Endoh M, Yang HT (2015). Ros generated during early reperfusion contribute to intermittent hypobaric hypoxia-afforded cardioprotection against postischemia-induced ca(2+) overload and contractile dysfunction via the jak2/stat3 pathway. Journal of molecular and cellular cardiology.

[22] Goodhead DT, Belli M, Mill AJ, Bance DA, Allen LA, Hall SC, Ianzani F, Simone G, Stevens DL, Stretch A (1992). Direct comparison between protons and alpha-particles of the same let: I. Irradiation methods and inactivation of asynchronous v79, hela and c3h 10t1/2 cells. International journal of radiation biology.

[23] Furusawa Y, Fukutsu K, Aoki M, Itsukaichi H, Eguchi-Kasai K, Ohara H, Yatagai F, Kanai T, Ando K (2000). Inactivation of aerobic and hypoxic cells from three different cell lines by accelerated (3)he-, (12)c- and (20)ne-ion beams. Radiation research.

[24] Adler V, Yin Z, Tew KD, Ronai Z (1999). Role of redox potential and reactive oxygen species in stress signaling. Oncogene.

[25] Simon HU, Haj-Yehia A, Levi-Schaffer F (2000). Role of reactive oxygen species (ros) in apoptosis induction. Apoptosis : an international journal on programmed cell death.

[26] Frankenberg D, Frankenberg-Schwager M, Garg I, Pralle E, Uthe D, Greve B, Severin E, Gohde W (2002). Mutation induction and neoplastic transformation in human and human-hamster hybrid cells: Dependence on photon energy and modulation in the low-dose range. Journal of radiological protection : official journal of the Society for Radiological Protection.

[27] Durante M, Furusawa Y, George K, Gialanella G, Greco O, Grossi G, Matsufuji N, Pugliese M, Yang TC (1998). Rejoining and misrejoining of radiation-induced chromatin breaks. Iv. Charged particles. Radiation research.

[28] Cardis E, Howe G, Ron E, Bebeshko V, Bogdanova T, Bouville A, Carr Z, Chumak V, Davis S, Demidchik Y, Drozdovitch V, Gentner N, Gudzenko N, Hatch M, Ivanov V, Jacob P, Kapitonova E, Kenigsberg Y, Kesminiene A, Kopecky KJ, Kryuchkov V, Loos A, Pinchera A, Reiners C, Repacholi M, Shibata Y, Shore RE, Thomas G, Tirmarche M, Yamashita S, Zvonova I (2006). Cancer consequences of the chernobyl accident: 20 years on. Journal of radiological protection : official journal of the Society for Radiological Protection.

[29] Scholz M, Ritter S, Kraft G (1998). Analysis of chromosome damage based on the time course of aberrations. International journal of radiation biology.

[30] Cosgrove GE, Upton AC, Congdon CC, Doherty DG, Christenberry KW, Gosslee DG (1964). Late somatic effects of x-radiation in mice treated with aet and isologous bone marrow. Radiation research.

[31] Ford DD, Paterson JC, Treuting WL (1959). Fetal exposure to diagnostic x rays, and leukemia and other malignant diseases in childhood. Journal of the National Cancer Institute.

[32] Shu XO, Potter JD, Linet MS, Severson RK, Han D, Kersey JH, Neglia JP, Trigg ME, Robison LL (2002). Diagnostic x-rays and ultrasound exposure and risk of childhood acute lymphoblastic leukemia by immunophenotype. Cancer epidemiology, biomarkers & prevention : a publication of the American Association for Cancer Research, cosponsored by the American Society of Preventive Oncology.

[33] Sancar A, Lindsey-Boltz LA, Unsal-Kacmaz K, Linn S (2004). Molecular mechanisms of mammalian DNA repair and the DNA damage checkpoints. Annual review of biochemistry.

[34] Prise KM, Pinto M, Newman HC, Michael BD (2001). A review of studies of ionizing radiation-induced double-strand break clustering. Radiation research.

[35] Frankenberg-Schwager M, Frankenberg D (1990). DNA double-strand breaks: Their repair and relationship to cell killing in yeast. International journal of radiation biology.

[36] Khanna KK, Jackson SP (2001). DNA double-strand breaks: Signaling, repair and the cancer connection. Nature genetics.

[37] Britt AB (1996). DNA damage and repair in plants. Annual review of plant physiology and plant molecular biology.

[38] Sundaresan M, Yu ZX, Ferrans VJ, Irani K, Finkel T (1995). Requirement for generation of h2o2 for platelet-derived growth factor signal transduction. Science.

[39] Gupta K, Kshirsagar S, Li W, Gui L, Ramakrishnan S, Gupta P, Law PY, Hebbel RP (1999). Vegf prevents apoptosis of human microvascular endothelial cells via opposing effects on mapk/erk and sapk/jnk signaling. Experimental cell research.

[40] Dimmeler S, Assmus B, Hermann C, Haendeler J, Zeiher AM (1998). Fluid shear stress stimulates phosphorylation of akt in human endothelial cells: Involvement in suppression of apoptosis. Circulation research.

[41] Aristizabal S, Caldwell WL, Avila J (1977). The relationship of time-dose fractionation factors to complications in the treatment of pituitary tumors by irradiation. International journal of radiation oncology, biology, physics.

[42] Calvo W, Hopewell JW, Reinhold HS, Yeung TK (1988). Time- and dose-related changes in the white matter of the rat brain after single doses of x rays. The British journal of radiology.

[43] Leach JK, Van Tuyle G, Lin PS, Schmidt-Ullrich R, Mikkelsen RB (2001). Ionizing radiation-induced, mitochondria-dependent generation of reactive oxygen/nitrogen. Cancer research.

[44] Kim GJ, Chandrasekaran K, Morgan WF (2006). Mitochondrial dysfunction, persistently elevated levels of reactive oxygen species and radiation-induced genomic instability: A review. Mutagenesis.

[45] Brookes PS, Yoon Y, Robotham JL, Anders MW, Sheu SS (2004). Calcium, atp, and ros: A mitochondrial love-hate triangle. American journal of physiology. Cell physiology.

[46] Tatsuta T, Langer T (2008). Quality control of mitochondria: Protection against neurodegeneration and ageing. The EMBO journal.

[47] Ashrafi G, Schwarz TL (2013). The pathways of mitophagy for quality control and clearance of mitochondria. Cell death and differentiation.

[48] Halestrap AP, Clarke SJ, Javadov SA (2004). Mitochondrial permeability transition pore opening during myocardial reperfusion--a target for cardioprotection. Cardiovascular research.

[49] Wallace DC (2013). A mitochondrial bioenergetic etiology of disease. The Journal of clinical investigation.

[50] Song M, Chen Y, Gong G, Murphy E, Rabinovitch PS, Dorn GW (2014). Super-suppression of mitochondrial reactive oxygen species signaling impairs compensatory autophagy in primary mitophagic cardiomyopathy. Circulation research.

[51] Detmer SA, Chan DC (2007). Functions and dysfunctions of mitochondrial dynamics. Nature reviews. Molecular cell biology.

[52] Twig G, Elorza A, Molina AJ, Mohamed H, Wikstrom JD, Walzer G, Stiles L, Haigh SE, Katz S, Las G, Alroy J, Wu M, Py BF, Yuan J, Deeney JT, Corkey BE, Shirihai OS (2008). Fission and selective fusion govern mitochondrial segregation and elimination by autophagy. The EMBO journal.

[53] Cribbs JT, Strack S (2007). Reversible phosphorylation of drp1 by cyclic amp-dependent protein kinase and calcineurin regulates mitochondrial fission and cell death. EMBO reports.

[54] Youle RJ, Narendra DP (2011). Mechanisms of mitophagy. Nature reviews. Molecular cell biology.

[55] Song M, Gong G, Burelle Y, Gustafsson AB, Kitsis RN, Matkovich SJ, Dorn GW (2015). Interdependence of parkin-mediated mitophagy and mitochondrial fission in adult mouse hearts. Circulation research.

[56] McBride H, Scorrano L (2013). Mitochondrial dynamics and physiology. Biochimica et biophysica acta.

[57] Shen YF, Tang Y, Zhang XJ, Huang KX, Le WD (2013). Adaptive changes in autophagy after ups impairment in parkinson’s disease. Acta pharmacologica Sinica.

[58] Ikeda Y, Shirakabe A, Brady C, Zablocki D, Ohishi M, Sadoshima J (2015). Molecular mechanisms mediating mitochondrial dynamics and mitophagy and their functional roles in the cardiovascular system. Journal of molecular and cellular cardiology.

[59] Huang H, Frohman MA (2009). Lipid signaling on the mitochondrial surface. Biochimica et biophysica acta.

[60] Zuchner S, Mersiyanova IV, Muglia M, Bissar-Tadmouri N, Rochelle J, Dadali EL, Zappia M, Nelis E, Patitucci A, Senderek J, Parman Y, Evgrafov O, Jonghe PD, Takahashi Y, Tsuji S, Pericak-Vance MA, Quattrone A, Battaloglu E, Polyakov AV, Timmerman V, Schroder JM, Vance JM (2004). Mutations in the mitochondrial gtpase mitofusin 2 cause charcot-marie-tooth neuropathy type 2a. Nature genetics.

[61] Dorn GW, Song M, Walsh K (2015). Functional implications of mitofusin 2-mediated mitochondrial-sr tethering. Journal of molecular and cellular cardiology.

[62] Ziviani E, Tao RN, Whitworth AJ (2010). Drosophila parkin requires pink1 for mitochondrial translocation and ubiquitinates mitofusin. Proceedings of the National Academy of Sciences of the United States of America.

[63] Chen Y, Csordas G, Jowdy C, Schneider TG, Csordas N, Wang W, Liu Y, Kohlhaas M, Meiser M, Bergem S, Nerbonne JM, Dorn GW, Maack C (2012). Mitofusin 2-containing mitochondrial-reticular microdomains direct rapid cardiomyocyte bioenergetic responses via interorganelle ca(2+) crosstalk. Circulation research.

[64] Ramonet D, Perier C, Recasens A, Dehay B, Bove J, Costa V, Scorrano L, Vila M (2013). Optic atrophy 1 mediates mitochondria remodeling and dopaminergic neurodegeneration linked to complex i deficiency. Cell death and differentiation.

[65] Song M, Dorn GW (2015). Mitoconfusion: Noncanonical functioning of dynamism factors in static mitochondria of the heart. Cell metabolism.

[66] Kuo CY, Chiu YC, Lee AY, Hwang TL (2015). Mitochondrial lon protease controls ros-dependent apoptosis in cardiomyocyte under hypoxia. Mitochondrion.

[67] Yin F, Sancheti H, Liu Z, Cadenas E (2015). Mitochondrial function in ageing: Coordination with signalling and transcriptional pathways. The Journal of physiology.

[68] Paglin S, Lee NY, Nakar C, Fitzgerald M, Plotkin J, Deuel B, Hackett N, McMahill M, Sphicas E, Lampen N, Yahalom J (2005). Rapamycin-sensitive pathway regulates mitochondrial membrane potential, autophagy, and survival in irradiated mcf-7 cells. Cancer research.

[69] Epperly MW, Sikora CA, DeFilippi SJ, Gretton JA, Zhan Q, Kufe DW, Greenberger JS (2002). Manganese superoxide dismutase (sod2) inhibits radiation-induced apoptosis by stabilization of the mitochondrial membrane. Radiation research.

[70] Yamamori T, Yasui H, Yamazumi M, Wada Y, Nakamura Y, Nakamura H, Inanami O (2012). Ionizing radiation induces mitochondrial reactive oxygen species production accompanied by upregulation of mitochondrial electron transport chain function and mitochondrial content under control of the cell cycle checkpoint. Free radical biology & medicine.

[71] Mikkelsen RB, Wardman P (2003). Biological chemistry of reactive oxygen and nitrogen and radiation-induced signal transduction mechanisms. Oncogene.

[72] Kim GJ, Fiskum GM, Morgan WF (2006). A role for mitochondrial dysfunction in perpetuating radiation-induced genomic instability. Cancer research.

[73] Yusuf SW, Sami S, Daher IN (2011). Radiation-induced heart disease: A clinical update. Cardiology research and practice.

[74] Hoppe RT (1997). Hodgkin’s disease: Complications of therapy and excess mortality. Annals of oncology : official journal of the European Society for Medical Oncology / ESMO.

[75] Swerdlow AJ, Higgins CD, Smith P, Cunningham D, Hancock BW, Horwich A, Hoskin PJ, Lister A, Radford JA, Rohatiner AZ, Linch DC (2007). Myocardial infarction mortality risk after treatment for hodgkin disease: A collaborative british cohort study. Journal of the National Cancer Institute.

[76] Aleman BM, van den Belt-Dusebout AW, Klokman WJ, Van’t Veer MB, Bartelink H, van Leeuwen FE (2003). Long-term cause-specific mortality of patients treated for hodgkin’s disease. Journal of clinical oncology : official journal of the American Society of Clinical Oncology.

[77] Cella L, Liuzzi R, Conson M, D’Avino V, Salvatore M, Pacelli R (2013). Multivariate normal tissue complication probability modeling of heart valve dysfunction in hodgkin lymphoma survivors. International journal of radiation oncology, biology, physics.

[78] Hayashi T, Morishita Y, Kubo Y, Kusunoki Y, Hayashi I, Kasagi F, Hakoda M, Kyoizumi S, Nakachi K (2005). Long-term effects of radiation dose on inflammatory markers in atomic bomb survivors. The American journal of medicine.

[79] Dunn MM, Drab EA, Rubin DB (1986). Effects of irradiation on endothelial cell-polymorphonuclear leukocyte interactions. Journal of applied physiology.

[80] Little MP, Gola A, Tzoulaki I (2009). A model of cardiovascular disease giving a plausible mechanism for the effect of fractionated low-dose ionizing radiation exposure. PLoS computational biology.

[81] Basavaraju SR, Easterly CE (2002). Pathophysiological effects of radiation on atherosclerosis development and progression, and the incidence of cardiovascular complications. Medical physics.

[82] Cella L, D’Avino V, Palma G, Conson M, Liuzzi R, Picardi M, Pressello MC, Boboc GI, Battistini R, Donato V, Pacelli R (2015). Modeling the risk of radiation-induced lung fibrosis: Irradiated heart tissue is as important as irradiated lung. Radiotherapy and oncology : journal of the European Society for Therapeutic Radiology and Oncology.

[83] Tribble DL, Barcellos-Hoff MH, Chu BM, Gong EL (1999). Ionizing radiation accelerates aortic lesion formation in fat-fed mice via sod-inhibitable processes. Arteriosclerosis, thrombosis, and vascular biology.

[84] Amromin GD, Gildenhorn HL, Solomon RD, Nadkarni BB (1964). The synergism of x-irradiation and cholesterol-fat feeding on the development of coronary artery lesions. Journal of atherosclerosis research.

[85] Marks LB, Yu X, Prosnitz RG, Zhou SM, Hardenbergh PH, Blazing M, Hollis D, Lind P, Tisch A, Wong TZ, Borges-Neto S (2005). The incidence and functional consequences of rt-associated cardiac perfusion defects. International journal of radiation oncology, biology, physics.

[86] Luo G, Xu X, Guo W, Luo C, Wang H, Meng X, Zhu S, Wei Y (2015). Neuropeptide y damages the integrity of mitochondrial structure and disrupts energy metabolism in cultured neonatal rat cardiomyocytes. Peptides.

[87] Dorn GW (2015). Cardiac regeneration -alchemy, science, and a wee bit of magic?. Journal of molecular and cellular cardiology.

[88] Yano M, Yamamoto T, Kobayashi S, Ikeda Y, Matsuzaki M (2008). Defective ca2+ cycling as a key pathogenic mechanism of heart failure. Circulation journal : official journal of the Japanese Circulation Society.

[89] Strong JP, Malcom GT, McMahan CA, Tracy RE, Newman WP, Herderick EE, Cornhill JF (1999). Prevalence and extent of atherosclerosis in adolescents and young adults: Implications for prevention from the pathobiological determinants of atherosclerosis in youth study. Jama.

[90] Dai DF, Chiao YA, Marcinek DJ, Szeto HH, Rabinovitch PS (2014). Mitochondrial oxidative stress in aging and healthspan. Longevity & healthspan.

[91] Dai DF, Karunadharma PP, Chiao YA, Basisty N, Crispin D, Hsieh EJ, Chen T, Gu H, Djukovic D, Raftery D, Beyer RP, MacCoss MJ, Rabinovitch PS (2014). Altered proteome turnover and remodeling by short-term caloric restriction or rapamycin rejuvenate the aging heart. Aging cell.

[92] Santulli G, Lombardi A, Sorriento D, Anastasio A, Del Giudice C, Formisano P, Beguinot F, Trimarco B, Miele C, Iaccarino G (2012). Age-related impairment in insulin release: The essential role of beta(2)-adrenergic receptor. Diabetes.

[93] Ciccarelli M, Chuprun JK, Rengo G, Gao E, Wei Z, Peroutka RJ, Gold JI, Gumpert A, Chen M, Otis NJ, Dorn GW, Trimarco B, Iaccarino G, Koch WJ (2011). G protein-coupled receptor kinase 2 activity impairs cardiac glucose uptake and promotes insulin resistance after myocardial ischemia. Circulation.

[94] Ciccarelli M, Santulli G, Pascale V, Trimarco B, Iaccarino G (2013). Adrenergic receptors and metabolism: Role in development of cardiovascular disease. Frontiers in physiology.

[95] Sorriento D, Franco A, Rusciano MR, Maione AS, Soprano M, Illario M, Iaccarino G, Ciccarelli M (2015). Good at heart: Preserving cardiac metabolism during aging. Current diabetes reviews.

[96] Cella L, Palma G, Deasy JO, Oh JH, Liuzzi R, D’Avino V, Conson M, Pugliese N, Picardi M, Salvatore M, Pacelli R (2014). Complication probability models for radiation-induced heart valvular dysfunction: Do heart-lung interactions play a role?. PloS one.

[97] Hardenbergh PH, Munley MT, Bentel GC, Kedem R, Borges-Neto S, Hollis D, Prosnitz LR, Marks LB (2001). Cardiac perfusion changes in patients treated for breast cancer with radiation therapy and doxorubicin: Preliminary results. International journal of radiation oncology, biology, physics.

[98] Yu X, Prosnitz RR, Zhou S, Hardenberg PH, Tisch A, Blazing MA, Borges-Neto S, Hollis D, Wong T, Marks LB (2003). Symptomatic cardiac events following radiation therapy for left-sided breast cancer: Possible association with radiation therapy-induced changes in regional perfusion. Clinical breast cancer.

[99] Gayed I, Gohar S, Liao Z, McAleer M, Bassett R, Yusuf SW (2009). The clinical implications of myocardial perfusion abnormalities in patients with esophageal or lung cancer after chemoradiation therapy. The international journal of cardiovascular imaging.

[100] Yaffe MP (1999). The machinery of mitochondrial inheritance and behavior. Science.

[101] Ainbinder A, Boncompagni S, Protasi F, Dirksen RT (2015). Role of mitofusin-2 in mitochondrial localization and calcium uptake in skeletal muscle. Cell calcium.

[102] Zoncu R, Efeyan A, Sabatini DM (2011). Mtor: From growth signal integration to cancer, diabetes and ageing. Nature reviews. Molecular cell biology.

[103] Guo P, Pi H, Xu S, Zhang L, Li Y, Li M, Cao Z, Tian L, Xie J, Li R, He M, Lu Y, Liu C, Duan W, Yu Z, Zhou Z (2014). Melatonin improves mitochondrial function by promoting mt1/sirt1/pgc-1 alpha-dependent mitochondrial biogenesis in cadmium-induced hepatotoxicity in vitro. Toxicological sciences : an official journal of the Society of Toxicology.

[104] Puigserver P, Spiegelman BM (2003). Peroxisome proliferator-activated receptor-gamma coactivator 1 alpha (pgc-1 alpha): Transcriptional coactivator and metabolic regulator. Endocrine reviews.

[105] Ong SB, Hall AR, Dongworth RK, Kalkhoran S, Pyakurel A, Scorrano L, Hausenloy DJ (2015). Akt protects the heart against ischaemiareperfusion injury by modulating mitochondrial morphology. Thrombosis and haemostasis.

[106] Pyakurel A, Savoia C, Hess D, Scorrano L (2015). Extracellular regulated kinase phosphorylates mitofusin 1 to control mitochondrial morphology and apoptosis. Molecular cell.

[107] Song M, Mihara K, Chen Y, Scorrano L, Dorn GW (2015). Mitochondrial fission and fusion factors reciprocally orchestrate mitophagic culling in mouse hearts and cultured fibroblasts. Cell metabolism.

[108] Chen M, Sato PY, Chuprun JK, Peroutka RJ, Otis NJ, Ibetti J, Pan S, Sheu SS, Gao E, Koch WJ (2013). Prodeath signaling of g protein-coupled receptor kinase 2 in cardiac myocytes after ischemic stress occurs via extracellular signal-regulated kinase-dependent heat shock protein 90-mediated mitochondrial targeting. Circulation research.

[109] Fusco A, Santulli G, Sorriento D, Cipolletta E, Garbi C, Dorn GW, Trimarco B, Feliciello A, Iaccarino G (2012). Mitochondrial localization unveils a novel role for grk2 in organelle biogenesis. Cellular signalling.

[110] Sorriento D, Fusco A, Ciccarelli M, Rungi A, Anastasio A, Carillo A, Dorn GW, Trimarco B, Iaccarino G (2013). Mitochondrial g protein coupled receptor kinase 2 regulates proinflammatory responses in macrophages. FEBS letters.

[111] Sorriento D, Ciccarelli M, Santulli G, Illario M, Trimarco B, Iaccarino G (2014). Trafficking grk2: Cellular and metabolic consequences of grk2 subcellular localization. Translational medicine @ UniSa.

[112] Zhang L, Jaswal JS, Ussher JR, Sankaralingam S, Wagg C, Zaugg M, Lopaschuk GD (2013). Cardiac insulin-resistance and decreased mitochondrial energy production precede the development of systolic heart failure after pressure-overload hypertrophy. Circulation. Heart failure.

